# Sub-Lethal 5-Fluorouracil Dose Challenges Planarian Stem Cells Promoting Transcriptional Profile Changes in the Pluripotent Sigma-Class Neoblasts

**DOI:** 10.3390/biom11070949

**Published:** 2021-06-26

**Authors:** Gaetana Gambino, Chiara Ippolito, Monica Evangelista, Alessandra Salvetti, Leonardo Rossi

**Affiliations:** 1 Department of Clinical and Experimental Medicine, University of Pisa, 56126 Pisa, Italy; gaetana.gambino@gmail.com (G.G.); chiara.ippolito@unipi.it (C.I.); leonardo.rossi@unipi.it (L.R.); 2 Institute of Clinical Physiology, CNR, 56124 Pisa, Italy; m.evangelista@ifc.cnr.it

**Keywords:** stem cells, 5-fluorouracil, planarian, sigma neoblasts

## Abstract

Under physiological conditions, the complex planarian neoblast system is a composite of hierarchically organized stem cell sub-populations with sigma-class neoblasts, including clonogenic neoblasts, endowed with larger self-renewal and differentiation capabilities, thus generating all the other sub-populations and dominating the regenerative process. This complex system responds to differentiated tissue demands, ensuring a continuous cell turnover in a way to replace aged specialized cells and maintain tissue functionality. Potency of the neoblast system can be appreciated under challenging conditions in which these stem cells are massively depleted and the few remaining repopulate the entire body, ensuring animal resilience. These challenging conditions offer the possibility to deepen the relationships among different neoblast sub-populations, allowing to expose uncanonical properties that are negligible under physiological conditions. In this paper, we employ short, sub-lethal 5-fluorouracil treatment to specifically affect proliferating cells passing through the S phase and demonstrate that S-phase slowdown triggers a shift in the transcriptional profile of sigma neoblasts, which reduces the expression of their hallmark *sox-P1*. Later, some cells reactivate *sox-P1* expression, suggesting that some neoblasts in the earlier steps of commitment could modulate their expression profile, reacquiring a wider differentiative potential.

## 1. Introduction

The planarian stem cell system, composed of sub-populations endowed with different self-maintenance, replicative, and differentiation potential, offers a unique opportunity to study the complex cross talk among stem cells themselves and between stem cells and differentiated cell compartments in an in vivo context. Planarian stem cells are referred to as neoblasts and share ultrastructural features, the ability to proliferate, and hallmarks in their transcriptional profile [1]. Neoblasts are involved in tissue homeostasis in intact organisms and, during regeneration, proliferate and accumulate below the wound, producing a blastema, where the missing body structures are reformed [2]. Among the different neoblast specific genes identified so far, *Schmidtea mediterranea smedwi1* [3] and its *Dugesia japonica* homologue *DjpiwiA* [4] are the most recognized markers to label a cell as a neoblast at present.

Recent data suggest that *smedwi-1*-positive cells are hierarchically organized, and a sub-population (the sigma-class neoblasts), showing elevated expression of *soxP-1*, *soxP-2*, *soxB-1*, *smad-6/7*, *inx-13*, *pbx-1*, *fgfr-4*, and *nlk-1*, is supposed to include clonogenic neoblasts (c-neoblasts), dominates the early wound response, and generates other neoblast subclasses. Sigma neoblasts are able to generate the zeta-class neoblasts of epidermal precursors. Transition in the transcriptional profile from sigma to zeta neoblasts has been suggested to begin directly upon entry into the S phase [5]. Once produced, the majority of the 2C cells derived from zeta-neoblast mitoses exit the cell cycle permanently [5] and are not able to perform multiple rounds of cell division and hence self-renewal [6]. However, the possibility that when appropriately challenged, zeta neoblasts might also self-renew and even transition into a sigma-neoblast state cannot be completely excluded [5]. This transition can be considered also for other still unidentified neoblast sub-classes, although significant proofs have never been provided. To provide a clue about the transition between neoblast sub-populations, we took advantage of our previous work in which we demonstrated the ability of a single 24 h (SDT) 5-fluorouracil (5FU) treatment at a dose of 6000 μM (SDT-6000) to dramatically reduce the number of *DjpiwiA*-positive cells, which completely disappeared 7 days after treatment, and to produce animal death in about 40 days after treatment [7]. In the same paper, we demonstrated that contrarily to SDT-6000, the lowest dose of 600 μM (SDT-600) did not produce a reduction in the *DjpiwiA* signal 7 days after treatment, and only some of the animals died at late times during the period of observation, suggesting that SDT-600 insult modulates stem cell behavior in some ways and pushing us to a deeper analysis of what happened in the treated animals in order to gain insight into planarian stem cell system complexity.

## 2. Materials and Methods

### 2.1. Animals, 5FU Treatment, and Regeneration Experiments

Planarians belonging to the species *D. japonica*, asexual strain GI [8], were raised at 18 °C, as previously described [9], and starved for 15 days before being used in the experiments. 5FU (Sigma-Aldrich, St. Louis, MO, USA, F6627) was diluted in dimethyl sulfoxide to produce a stock solution, as previously described [7]. Treatments were performed by diluting 5FU stock solution in planarian water to a final concentration of 600 or 6000 µM. Next, 15 mL of 5FU solution was used to treat 45 animals in 10 cm Ø Petri dishes for 24 h. After treatment, 5FU solution was removed and the animals were washed seven times with fresh planarian water. Regenerating fragments were obtained by transection between auricles and the pharynx. Regenerative performance (blastema area/regenerating fragment area) was calculated for regenerating tails 4 days after cutting, as previously described [10].

### 2.2. Whole Mount In Situ Hybridization and Post-Hybridization Immunofluorescence

DNA templates for *DjpiwiA*, *DjPiwi-1*, *Djnos*, *DjsoxP-1*, *Djgata4-5-6*, *DjP53*, *DjNB.21.11.e*, *DjAGAT2*, *Djinnexin1*, *DjSyt*, and *DjCIP29* were obtained, as previously described [7,9,10,11,12,13,14,15]. The DNA template for *DjEGR-1* (gi|393820293), the *D. japonica* homologue of the zeta-class neoblasts, *Smed-egr-1* [5], was obtained by RT-PCR using the forward primer 5′ ACGAAGGAAGATAATAAAAGTCGAG 3′ and the T7-adapted reverse primer 5′ CGGATATAATACGACTCACTATAGGGGGGAACTACTTTTATCACTAAATGG 3′. DNA templates for *DjIFb* and *DjCTL* were obtained using the following forward and T7-adapted reverse primers: 

*DjIFb* F: 5′ GGGGTAAAGAAACTGCCAGA 3′

*DjIFb R*: 5′ CGGATATAATACGACTCACTATAGGGTCTTCTTCTAGCGAGCTCAAAC 3′

*DjCTL* F: 5′ AGTATGAAATTACCAGTGATCG 3′

*DjCTL* R: 5′ CGGATATAATACGACTCACTATAGGGGTTTACAGGATCACAGATGAC 3′

Probe synthesis and whole-mount in situ hybridization were performed according to [16]. Densitometry analysis of the hybridization signal intensity was performed according to [7] on five animals for each experimental class using ImageJ software [17]. A background adjustment was applied by subtracting the mean gray value recorded from the unstained pharynx region of each animal. Some hybridized specimens were paraffin-embedded, cut in 5 µm sections, stained with Direct Red 80 (Sigma-Aldrich, 365548-5G), and counterstained with Fast Green (Sigma-Aldrich, F7252), as previously described [9]. Post-hybridization immunostaining with anti-phosphorylated histone-H3 (H3p, Sigma-Aldrich, 06-570) antibody was performed, as previously described [9]. Post-hybridization immunostaining with anti-synapsin (Developmental Studies Hybridoma Bank) antibody was essentially performed as described by [15] using an HRP-conjugated anti-mouse secondary antibody (Biovision, Inc., Milpitas, CA, USA) at 1:1000 dilution. The signal was revealed by tyramide amplification, as described in the BrdU detection chapter. For each specimen, a single composite image, with optical sectioning every 2 µm of the whole sample, was captured by both tile scan and zeta stack acquisition mode of a TCS SP8 confocal microscope (Leica Microsystems CMS, Wetzlar, Germany). At least two independent experiments were performed for each experimental dataset to verify the consistency of the results. The number of H3p-positive cells was recorded in composite images by using the find maxima option of ImageJ software.

### 2.3. Bromodeoxyuridine Labeling and Immunofluorescence on Tissue Sections

BrdU (Sigma-Aldrich, B9285) was diluted and injected into the planarian body, as previously described [7]. Twenty-four hours after BrdU injection, the animals were killed in 2% HCl in 5/8 Holtfreter for 5 min at 4 °C, fixed in relaxant solution, and paraffin-embedded, as described in [15]. Next, 6-µm-thick tissue sections were placed on SuperFrost Plus slides, dewaxed in xylene, rehydrated through a graded series of ethanol dilutions, and rinsed for 5 min in phosphate-buffered saline plus 0.1% Triton X-100 (PBST01). After equilibration in phosphate-buffered saline plus 0.3% Triton X-100 (PBST03) at 37 °C for 5 min, tissue sections were permeabilized by proteinase K (Sigma-Aldrich, 1.24568) treatment at 37 °C (5 µg/mL in PBST03) for 7 min and then quickly washed two times in PBST01. DNA was then denatured for 5 min in 1N HCl in PBST01 at 55 °C. HCl was then removed by three washes (10 min each) in PBST01, and tissue sections were blocked in 10% fetal bovine serum in PBST01 (blocking solution) for 1 h. Slides were probed with anti-BrdU antibody (anti-BrdU B44, BD Biosciences) at 1:50 dilution in blocking solution for 1 h and then washed three times (10 min each) in phosphate-buffered saline plus 0.5% Triton X-100 (PBST05) to remove unbound antibodies. Bound primary anti-BrdU antibodies were then detected by incubating slides with HRP-conjugated anti-mouse secondary antibody (Biovision, Inc.) at 1:1000 dilution in blocking solution for 1 h. After three washes in PBST05 (10 min each), the slides were equilibrated in TSA buffer (0.1 M sodium borate, 2 M NaCl; pH 8.5), and the signal was revealed by incubating for 15 min with tyramide-conjugated N-hydroxy-succinimydyl esters (FAM) prepared and diluted following the detailed protocol described in [18]. After three washes (10 min each) in PBST01, HRP was inactivated by incubating slides in 100 mM sodium azide in PBST01 for 45 min and then washed overnight at 4 °C in PBST01. The next day, the slides were blocked in blocking solution and probed with anti-phosphorylated histone-H3 antibody at 1:500 dilution in blocking solution for 1 h. After PBST01 washing, bound primary antibodies were detected by using HRP-conjugated anti-rabbit secondary antibody (Biovision, Inc.) at 1:1000 dilution and N-hydroxy-succinimydyl esters (TAMRA), as described above. Five animals for each experimental class were immunostained, and the number of BrdU- and H3p-positive cells were recorded in 10 tissue sections for each animal (5 behind and 5 below the pharynx region). Two independent experiments were performed for each experimental dataset to verify the consistency of the results.

### 2.4. Cytofluorimetric Analysis of DNA Content

DNA content was evaluated by propidium iodide (PI) staining, followed by flow cytometric analysis using a modified version of the protocol described in [19]. In particular, a pool of 4 planarians was placed in 300 μL of 0.1 M citric acid plus 0.5% Tween-20 for 10 min at room temperature, vortexed for 10 s, and neutralized by adding 1 mL of 0.4 M sodium phosphate dibasic containing 6 μg/mL of RNAse A. After 10 min incubation at room temperature, PI was added at a final concentration of 10 μg/mL. Cell suspensions were filtered through a 50 μm nylon mesh, stained for 1 h, and analyzed on a linear scale by using the ACCURI C6 cytofluorimeter (Becton Dickinson, Franklin Lake, NJ, USA) with doublet discrimination. Finally, 10^5^ events per sample were acquired, and the percentage of cells in the G1-, S-, and G2-phase fractions was determined according to PI intensity (FL2-A). Five independent samples for each experimental class were analyzed. 

### 2.5. TUNEL Assay

TUNEL assay was performed using the ApopTag^®^ Red In Situ Apoptosis Detection Kit (EMD Millipore Corp., Burlington, MA, USA) following the manufacturer’s instructions and adaptation described in [9]. Five planarians were analyzed for each experimental condition. For each specimen, a single composite image, with optical sectioning every 2 µm of the whole sample, was captured by both tile scan and zeta stack acquisition mode of a TCS SP8 confocal microscope (Leica Microsystems CMS). Two independent experiments were performed for each experimental dataset to verify the consistency of the results. The number of apoptotic cells was evaluated using the find maxima option of ImageJ software.

### 2.6. Statistical Analysis

The non-parametric, two-tailed Mann–Whitney *U*-test was applied across each experiment to evaluate the statistical significance of differences for each treatment group versus the untreated control group. Differences were considered statistically significant if the Mann–Whitney *U*-test retrieved a *p*-value below 0.05. For densitometry analysis of the *DjsoxP-1*, *DjpiwiA*, *DjNB.21.11.e*, and *DjEGR-1* hybridization signal, whole-mount H3p immunostaining and TUNEL assay, the analysis was performed considering each single animal as an independent experimental replicate (*n* = 5). For BrdU and H3p immunolabeling, the mean number of positive cells recorded from the 10 tissue sections analyzed for each animal was considered as an independent experimental replicate (*n* = 5). 

## 3. Results

### 3.1. Analysis of Stem Cell Markers Following SDT-600 Treatment

**Figure 1:**
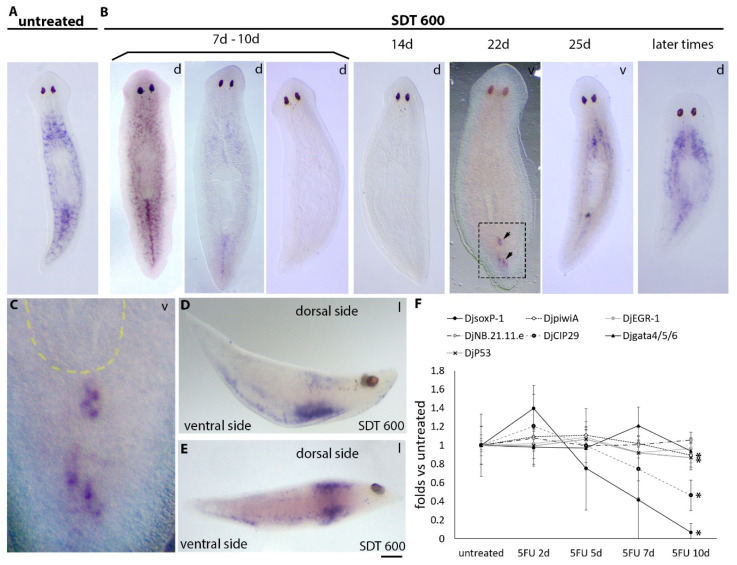
*DjsoxP-1* expression visualized by whole-mount in situ hybridization in intact untreated controls and SDT-600 animals. (**A**) Representative image of *DjsoxP-1* in situ hybridization in an untreated control. (d) Dorsal view. (**B**) Representative images of *DjsoxP-1* in situ hybridization in 5FU-treated animals at different times after treatment. Different images are shown for the temporal window between 7 and 10 days to represent the inter-experimental variability. Arrows indicate the most limited expression found for *DjsoxP-1* in SDT-600 animals 22 days after treatment. (d) Dorsal view; (v) ventral view. (**C**) Magnification of the boxed region of an SDT-600 animal 22 days after treatment shown in (**B**). (v) Ventral view. Yellow dashed line indicates the pharynx. (**D**,**E**) Lateral view (l) of some representative animals sacrificed at later times after SDT-600 treatment and hybridized with *DjsoxP-1*. The two animals showed different levels of repopulation of the dorsal side. Scale bar corresponds to 400 µm in (**A**,**B**), 80 µm in (**C**), and 200 µm in (**D**,**E**). (**F**) Graph depicting densitometry analysis of hybridized animals at different time points during the first 10 days of treatments. Each point is the mean value ± SD of the data obtained in at least two different experimental replicates of normalized versus untreated controls, in which the arbitrary value of 1 has been attributed. The high SD of the *DjsoxP-1* value 7 days after treatment is attributable to inter-experimental differences in the time at which the expression of this transcript starts to decrease. Statistically significant differences (* *p* < 0.05) are indicated only for time points at which they have been verified in all the experimental sets; only *DjsoxP-1*, *DjpiwiA*, *DjCIP29*, and *DjP53* showed statistically significant variations.

**Figure 2:**
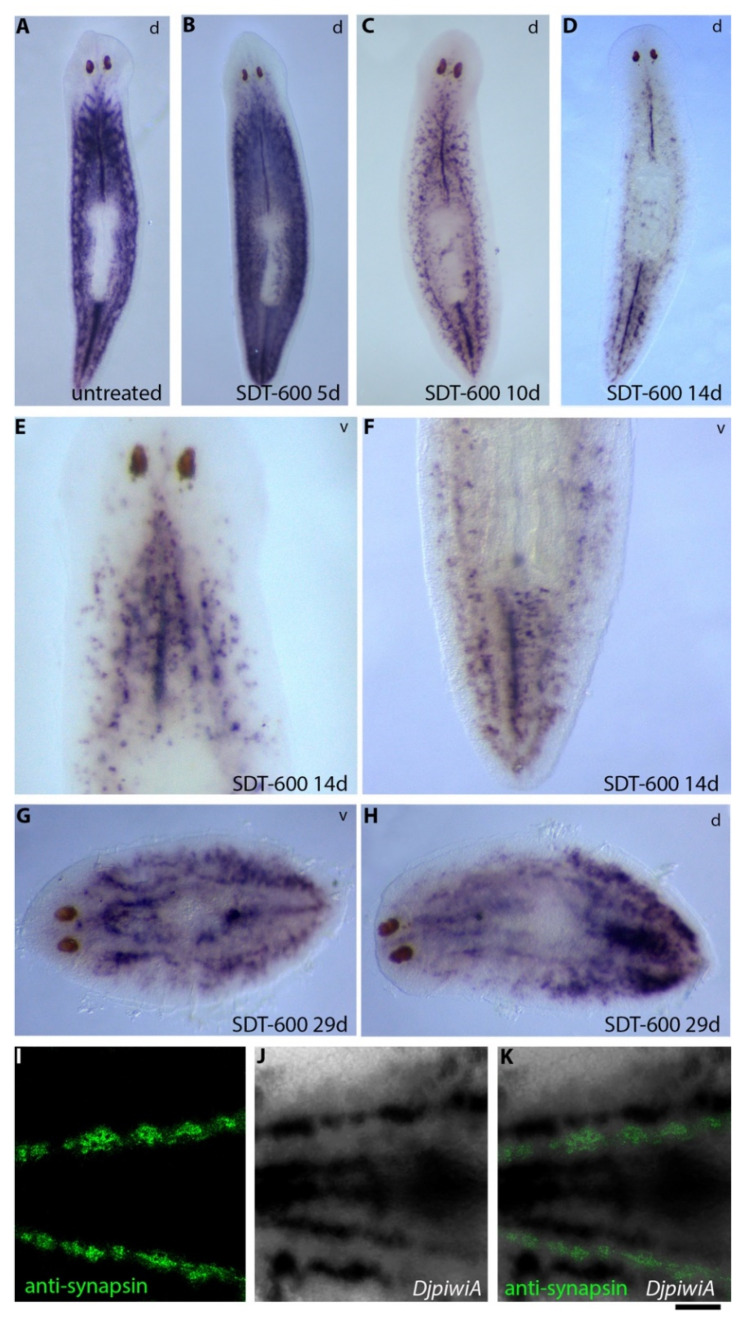
*DjpiwiA* expression visualized by whole-mount in situ hybridization in intact SDT-600 animals and untreated controls. (**A**) Representative image of a control untreated animal in dorsal (d) view. (**B**) Representative image of an SDT-600 animal 5 days after treatment. (**C**) Representative image of an SDT-600 animal 10 days after treatment in dorsal (d) view. (**D**–**F**) Representative images of an SDT-600 animal 14 days after treatment in dorsal (d) and ventral (v) views. (**G**,**H**) Representative images of an SDT-600 animal 29 days after treatment in dorsal (d) and ventral (v) views. (**I**–**K**) Post-hybridization immunostaining with anti-synapsin antibody that reveals proximity between anti-synapsin-positive ventral nerve cords (green) and *DjpiwiA* hybridization signal (dark brown). Scale bar corresponds to 500 µm in (**A**–**D**), 110 µm in (**E**–**F**), 200 µm in (**G**–**H**), and 80 µm in (**I**–**K**).

With the aim to understand stem cell fate after SDT-600 treatment, we analyzed the expression of the sigma-class neoblast marker *DjsoxP-1* at different time points in SDT-600 animals in comparison to SDT-6000 animals. As shown in Figure S1, in SDT-6000 animals, *DjsoxP-1* expression became completely and definitely undetectable from the seventh day after treatment. On the contrary, *DjsoxP-1* showed modulated expression at different time points in SDT-600 animals (Figure 1). In particular, *DjsoxP-1* dramatically dropped between 7 and 10 days after treatment with inter-experimental differences (Figure 1A,B,F and Table S1) and became undetectable, by whole-mount in situ hybridization, at 14 days after treatment in most of the analyzed animals (39/50). *DjsoxP-1* transcripts were, in any case, always detectable in cDNA obtained from 14-day-treated organisms, suggesting that cells that normally express high levels of this transcript (*DjsoxP-1^high^* cells) downregulate its transcription under the limit of resolution of the in situ hybridization technique, becoming *DjsoxP-1^low^* cells. Surprisingly, starting from about 22 days after treatment, *DjsoxP-1^high^* cells reappeared in the bodies of most of the specimens (Figure 1B,C). Animals showed asynchronous reactivation of *DjsoxP-1* expression, and Figure 1B shows the typical pattern of the animals with the most limited expression of this transcript that we found analyzing several hybridized specimens (*n* = 50). In this case, *DjsoxP-1^high^* cells were exclusively detectable in the tail region, close to the ventral surface, in the mesenchyme located between the two posterior gut branches, with recurrent accumulation immediately posterior to the pharynx opening (arrows in Figure 1B,C). As shown in Figure S2 and Table S1, other animals showed a wider expression territory, including the same mesenchymal region, with the addition of some cells always close to the ventral surface, which, in most cases, were organized in rows that reached the anterior part of the animal body. About 25 days after treatment, in most of the animals, *DjsoxP-1^high^* cells remained in the caudal region, with a similar expression pattern as previously described, and in the rest of the ventral surface of the animals appeared mainly organized in rows (Figure 1B). Up to this time, no *DjsoxP-1^high^* cells were detected in the dorsal mesenchyme. However, later, the expression pattern profoundly changed and *DjsoxP-1^high^* cells appeared in the dorsal mesenchyme (Figure 1B,D,E and Table S1), with a distribution reminiscent that of untreated organisms. Differently from *DjsoxP-1^high^* cells, *DjpiwiA*-positive cells never completely disappeared from the animal body (Figure 1F and Figure 2). Indeed, 10 days after treatment *DjpiwiA* expression started a significant reduction (Figure 1F and Figure 2C), and only after 14 days, a strong decrease in *DjpiwiA*-positive cells was observed and remnant cells distributed in the dorsal side of the animals, as for the previously described TRSC (Figure 2D), while at the ventral side, they appeared widely distributed and preferentially accumulated in rows (Figure 2E,F). This distribution remained basically unchanged for both animal sides up to 25 days after treatment, at which time the distribution in rows at the ventral side became more evident (Figure S3). Later, the *DjpiwiA* signal became increasingly stronger at the ventral side (Figure 2G), and repopulation of the dorsal side of the animals became evident 29 days after treatment (Figure 2H). In all these cases, most of the *DjsoxP-1* and *DjpiwiA* ventral expression appeared organized in rows, which followed the ventral nerve cords, as demonstrated by using the central-nervous-system-specific antibody anti-synapsin (Figure 2I–K). Similarly to *DjsoxP-1*, the expression of a second gene, *CIP29*, also involved in sigma-class neoblast maintenance [20], was significantly downregulated 10 days after treatment, although to a lesser extent than *DjsoxP-1* (Figure 1F).

**Figure 3:**
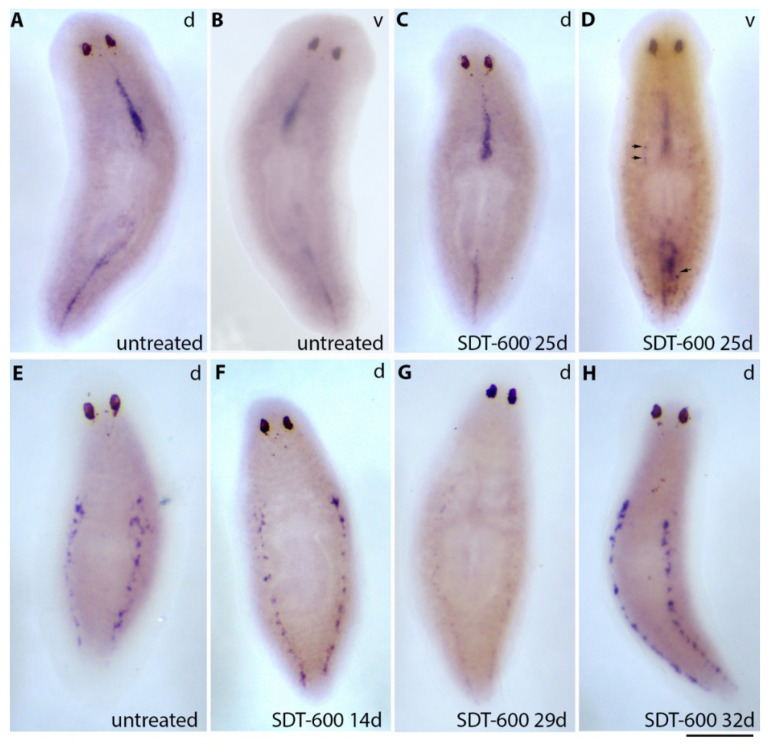
*Djpiwi-1* and *Djnos* expression visualized by whole-mount in situ hybridization in intact SDT-600 animals and untreated controls. (**A**,**B**) Representative images of a control untreated animal hybridized with a *Djpiwi-1* probe in dorsal (d) and ventral (v) views. (**C**,**D**) Representative images of an SDT-600 animal hybridized with a *Djpiwi-1* probe 25 days after treatment in dorsal (d) and ventral (v) views. Arrows indicate novel *Djpiwi-1* -positive cells at the ventral side of the animal. (**E**) Representative image of a control untreated animal hybridized with a *Djnos* probe in dorsal (d) view. (**F**–**H**) Representative images of an SDT-600 animal hybridized with a *Djnos* probe 14, 29, and 32 days after treatment. Scale bar corresponds to 500 µm.

*DjPiwi1* [13] is one of the five piwi paralogues found in *D. japonica* whose expression is exclusively detectable by in situ hybridization in a cord of neoblast-like cells at the dorsal midline (Figure 3A). *DjPiwi1*-positive cells are resistant to 5FU treatment, being part of the previously described TRSC [7], and the dorsal stripe of *DjPiwi1*-positive cells remained unaltered at all the analyzed time points. Strikingly, novel *DjPiwi1*-positive cells appeared, 22–25 days after treatment, in the ventral side of the animal (Figure 3D), a territory in which this marker is undetectable, by in situ hybridization, in untreated organisms (Figure 3B). These cells were mainly detectable in the tail region, with a distribution similar to that described for the *DjsoxP-1* cells, although we have no proof they are the same cells, and also extended to the more anterior part of the animals, always in rows (see arrows in Figure 3D). At the dorsal side of the animal, *DjPiwi1* was still detectable in an unmodified pattern in the dorsal midline cord (Figure 3C). *DjPiwi1* expression in ventral cells was transient, and at later stages, no more DjPiwi1 signals were detectable at the ventral side of the animal (data not shown). The repopulation process did not include *Djnos*-positive germline progenitors, which are normally organized in dorsolateral clusters [21], and 14 days after 5FU treatment, *Djnos* expression was unchanged with respect to controls (Figure 3E,F). Later, in the temporal window from 22 to 29 days after treatment, *Djnos*-positive clusters of cells dramatically reduced in size and signal intensity (Figure 3G). A completely restored pattern was reacquired about 32 days after treatment (Figure 3H). During all this process, no *Djnos*-positive cells appeared at the ventral side of the animal (data not shown). 

### 3.2. Analysis of Cell Cycle Progression and Apoptosis Following SDT-600 Treatment

**Figure 4:**
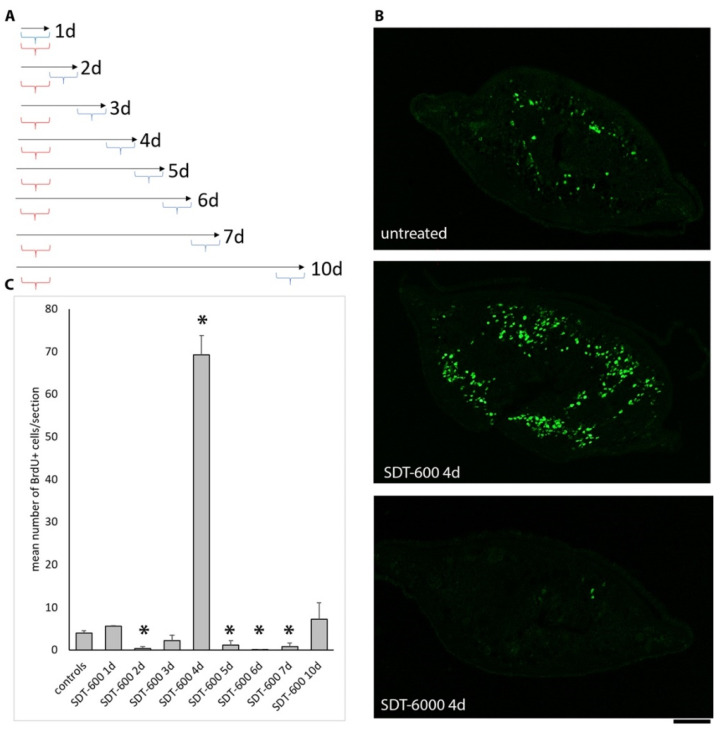
Analysis of BrdU incorporation. (**A**) Graph depicting the experimental setup. Red brackets indicate 5FU treatment, and blue brackets indicate the BrdU labeling time lapse. (**B**) Representative images of BrdU immunostaining on sections. (**C**) Histogram depicting the mean number of BrdU-positive cells counted during the first 10 days of treatment according to the experimental setup depicted in (**A**). Each bar is the mean value ± SD from five independent animals. Asterisks indicate a statistically significant difference (* *p* < 0.01) evaluated by the non-parametric, two-tailed Mann–Whitney *U*-test with respect to untreated controls. Scale bar corresponds to 125 µm.

**Figure 5:**
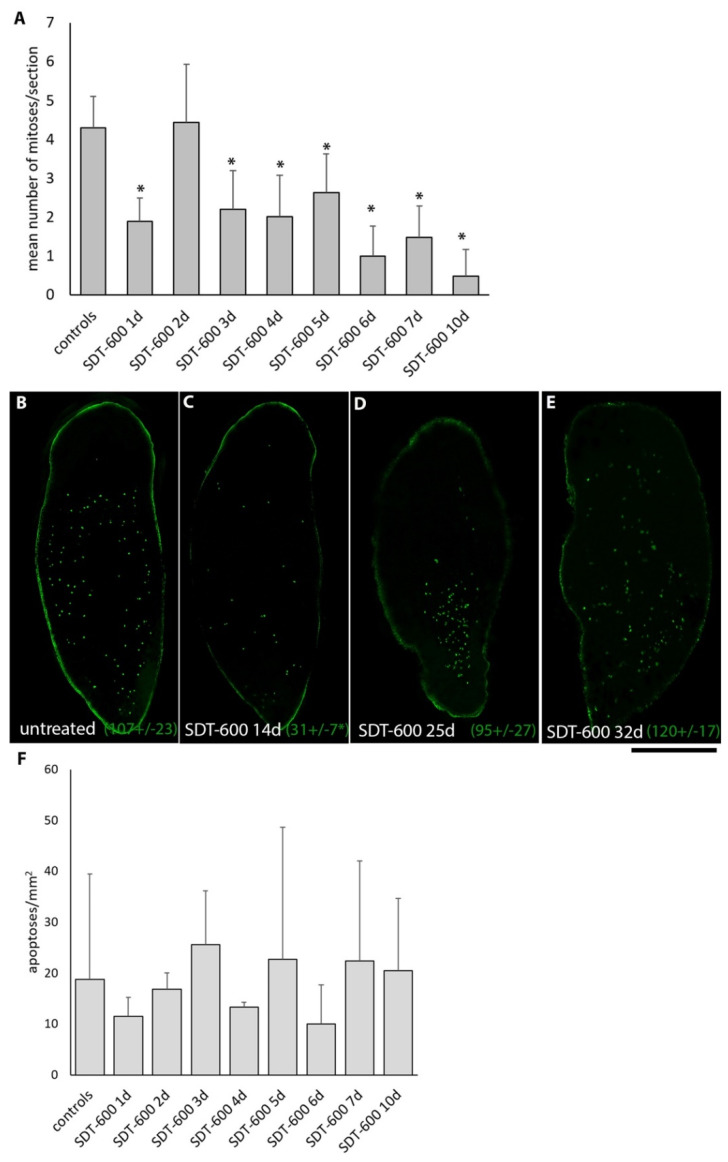
Analysis of mitotic and apoptotic cells. (**A**) Histogram depicting the mean number of H3p-positive cells counted during the first 10 days of treatment. Each bar is the mean value ± SD from five independent animals. Asterisks indicate a statistically significant difference (* *p* < 0.01) evaluated by the non-parametric, two-tailed Mann–Whitney *U*-test versus untreated controls. (**B**–**E**) Representative images of whole-mount H3p immunostaining. Mean number of H3p-positive cells ± SD are indicated in brackets; asterisk indicates a statistically significant difference (* *p* < 0.05) evaluated by the non-parametric, two-tailed Mann–Whitney *U*-test versus untreated controls. (**F**) Histogram depicting the mean number of TUNEL-positive cells counted during the first 10 days of treatment. Each bar is the mean value ± SD from five independent animals. No significant differences were evaluated by the non-parametric, two-tailed Mann–Whitney *U*-test versus untreated controls. Scale bar corresponds to 500 µm.

At day 7 after treatment, *DjsoxP-1*-positive cells started reducing, while *DjpiwiA*- and *DjCIP29*-positive cells were still present, supporting the idea that cell damage caused by 5FU treatment may trigger a transcriptional profile change and not bring cells to death. To verify this hypothesis, we monitored cell cycle progression and cell death during the first 10 days after SDT-600 treatment. Two days after SDT-600 treatment, extremely rare BrdU-positive cells were detectable in treated animals (Figure 4C). However, in a period between days 3 and 5 (with inter-experimental variability), a surprisingly massive BrdU incorporation peak was monitored with respect to untreated controls (Figure 4B,C). This was exclusive for SDT-600 treatment, while SDT-6000 treatment almost completely abolished BrdU incorporation except for a few resistant cells (Figure 4B). The number of BrdU-positive cells dropped to the basal level (below the control) in the following days and restarted to grow 10 days after treatment (Figure 4C). The number of H3p mitotic cells immediately dropped after SDT-600 treatment, and although some mitotic cells were always detectable in the animal body, they remained lower than controls up to 25 days after treatment (Figure 5A–C), when the mitotic cell number increased at the ventral side of the animal, especially in the tail region (Figure 5D). Later, mitotic activity became widely distributed at the ventral side of the animal (Figure 5E), and no significant accumulation of mitotic cells was observed at the dorsal surface (data not shown). To confirm the increase in S-phase cells monitored by BrdU incorporation a few days after SDT-600 treatment, we also monitored cell cycle distribution of cell dispersion obtained dissociating entire planarians. Four days after treatment, a slight significant increase in S-phase cells was recorded (Figure S4). Thus, SDT600 treatment profoundly modulates the stem cell cycle. However, differently from what was observed for the higher 6000 μM dose [7], no significant increase in cell death was detectable in the same period of time following SDT-600 treatment (Figure 5F). 

### 3.3. Correlation between the Transcriptional Change in the DjsoxP-1 Level and the Expression of Lineage Differentiation Markers

Several data are available about epidermal lineage commitment and differentiation in planarians [5,22,23]. Committed epidermal-lineage-restricted neoblasts, the zeta neoblasts, characterized by the expression of a group of molecular markers, including the *Egr-1* transcript, divide to produce the early epidermal progeny, post-mitotic progenitors characterized by the expression of *NB.21.11.e*, which then gradually differentiate into multiple epidermal cell types. Thus, the analysis of epidermal lineage differentiation markers makes it possible to monitor changes in the differentiation dynamics from pluripotent neoblasts to specialized cells. Quantitative analysis of hybridized specimens revealed that neither *DjEGR-1* nor *DjNB.21.11.e* reduces its expression following 5FU treatment up to 10 days (Figure 1F). On the contrary, a slight significant reduction was measured 10 days after treatment in *DjP53*, a transcription factor thought to be involved in sigma-to-zeta neoblast transition [24]. Several other fate-specific transcription factors have been identified to label fate-committed neoblasts; among them, we analyzed the expression of *Djgata4/5/6*, the *D. japonica* homologue of the three mammalian transcription factors GATA-4, GATA-5, and GATA-6, which are expressed in neoblasts committed toward an intestinal fate [25]. As for epidermal-committed neoblasts, *Djgata4/5/6*-positive cells also maintain an unaltered expression level during the first 10 days after treatment (Figure 1F).

### 3.4. Analysis of Regenerative Performances and DjsoxP-1 Reactivation Following SDT-600 Treatment

**Figure 6:**
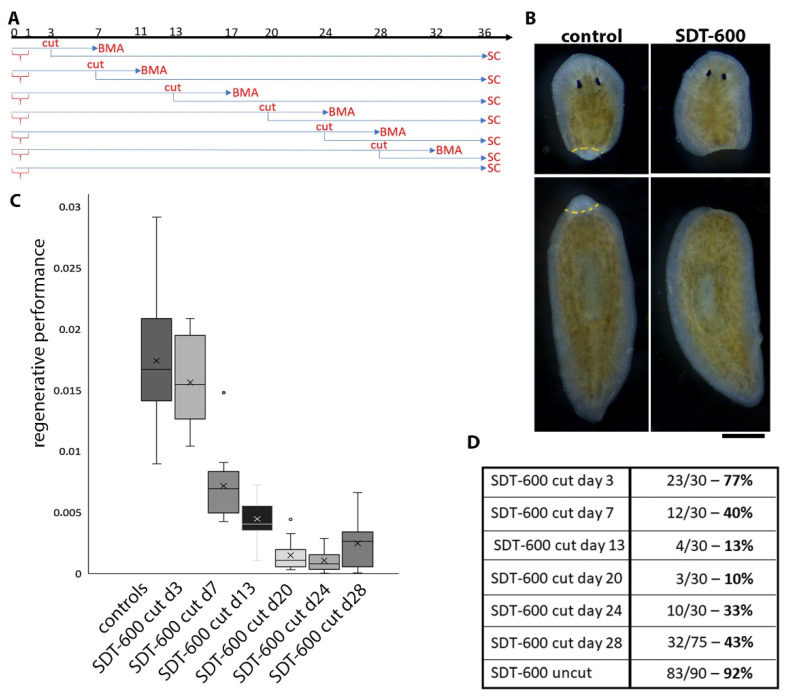
Analysis of regenerative performance and survival rate. (**A**) Graph depicting the experimental setup. Red brackets indicate 5FU treatment. BMA = blastema morphometric analysis; SC = survival count. (**B**) Representative images of head and tail pieces cut 13 days after 5FU treatment and fixed for morphometric analysis of blastema size at the fourth day of regeneration. (**C**) Box plot showing the regenerative performance (blastema area/regenerating fragment area) of animals cut according to the experimental plan described in (**A**); horizontal lines in the box plot indicate the median values; X = mean value. (**D**) Table showing the percentage of alive animals at day 36 from treatment. Scale bar corresponds to 500 µm.

**Figure 7:**
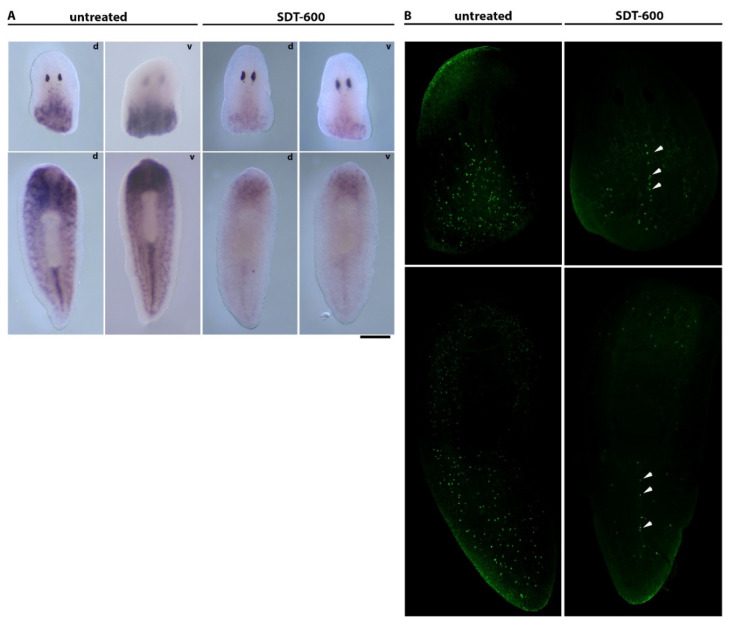
Analysis of *DjsoxP-1* expression and mitotic activity after 5FU treatment. (**A**) Representative images in dorsal (d) and ventral (v) views of *DjsoxP-1* in situ hybridization in 2-day-old regenerating control and treated animals sacrificed 15 days after 5FU treatment. (**B**) Representative images of H3p immunostaining in 2-day-old regenerating control and treated animals sacrificed 15 days after 5FU treatment. Arrows indicate mitosis in the dorsal midline cord. Scale bar corresponds to 400 µm in (**A**) and 130 µm in (**B**).

SDT-600 treatment strongly reduced regenerative performance (blastema size/body size) in animals cut at different times, both before *DjsoxP-1^high^* cell disappearance and after their reappearance. Indeed, although *DjsoxP-1* expression and proliferative activity were restored in the animal body, starting from about 22–25 days after treatment, planarians remained unable to regenerate up to 28 days after treatment, when a timid regenerative attempt could be monitored (Figure 6A,B). Ninety days after treatment, some animals self-divided and regenerated. Cutting animals during the rescue process severely worsened their survival rate in comparison to what was observed for uncut animals (Figure 6D). Thirteen days after SDT-600 treatment, no *DjsoxP-1^high^* cells were detectable in intact organisms. We wondered whether tissue wounding might accelerate/stimulate *DjsoxP-1^high^* cell reappearance. Thus, we cut pieces of the animals’ tails and heads at day 13 after treatment and sacrificed them for in situ hybridization 2 days later. Brand-new *DjsoxP-1* expression was detectable at both ventral and dorsal sides of the animals, close to the wound surface (Figure 7A), and the dorsal signal in this case was wider and stronger than the ventral one. In tail pieces, a faint *DjsoxP-1* expression, at the borderline of the detection limit, was also visible in cords in the tail region. To evaluate whether the new *DjsoxP-1^high^* cells were endowed with proliferative activity, we monitored H3p immunostaining in control and SDT-600 animals. As shown in Figure 7B, a reduced number of mitotic cells was detectable in SDT-600 organisms with respect to untreated controls. Moreover, the few H3p-positive cells were almost exclusively located at the dorsal side of the animals, as for controls, and most of them were aligned in a row in the dorsal midline cord (see arrows in Figure 7B). In tail pieces, we also monitored activation of mitosis far from the wound, in a region in which *DjsoxP-1^high^* cells were not detected. A second SDT-600 treatment at day 5 after the first dose dramatically abolished *DjsoxP-1^high^* cell reappearance after wounding (Figure S5).

### 3.5. Analysis of Differentiated Tissues at Late Times after SDT-600 Treatment

**Figure 8:**
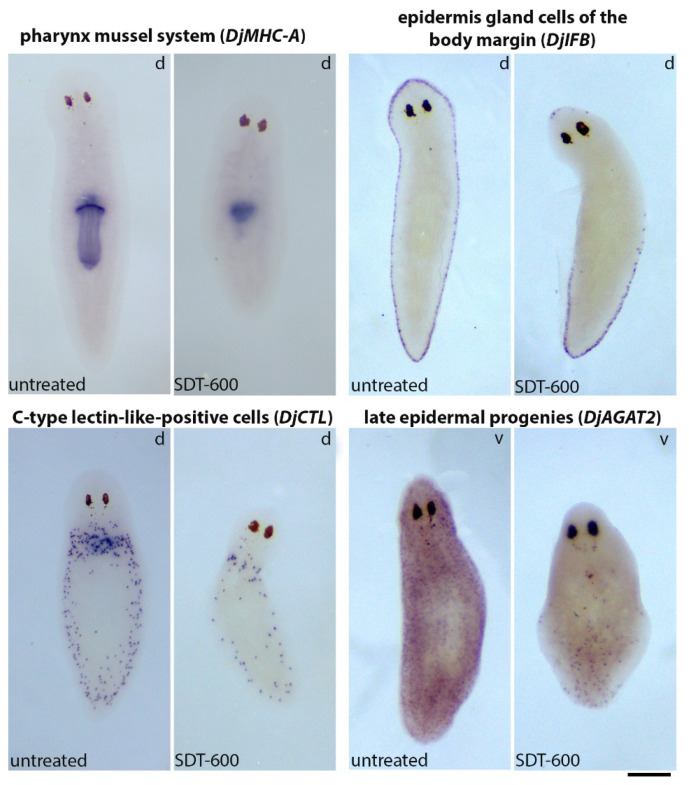
Analysis of the expression of differentiated tissue markers in untreated controls and in SDT-600 animals sacrificed 26 days after treatment. Scale bar corresponds to 500 µm.

To confirm that at late times after SDT-600 treatment, tissue turnover is compromised, we analyzed the expression of different cell tissue markers, including the gastrodermal system (*Djinnexin1*) [26], epidermis gland cells of the body margin (*DjIFb*) [27], the central nervous system (*Djsyt*) [28], the pharynx mussel system (*DjMHC-A*) [29], late epidermal progenies (*DjAGAT2*) [9], and C-type lectin-like (*DjCTL*)-positive cells of the innate immune system [30]. Then, 26 days after treatment, except for the gastrodermal system and the central nervous system, which did not appear macroscopically different between controls and SDT-600 animals (data not shown), we detected important changes in all the other analyzed tissues (Figure 8). In particular, the pharynx size was dramatically reduced; epidermis gland cells of the body margin were no more observable in the anterior part of the animals; the number of C-type lectin-like-positive cells was strongly reduced, and only a few late epidermal progenies were still detectable. We also took advantage of the ability of Direct Red 80 dye to exclusively stain, in post-hybridization specimens, some parapharyngeal glands. These gland cells were abundant in both ventral and dorsal sides of control animals in sections obtained immediately above the pharynx opening (Figure S6). In addition, 22 days after 5FU treatment, at the same body level, several gland cells were still observable, although most of them appeared pale and empty (Figure S6). Only a few small gland cells were detectable in most of the animals 32 days after treatment (Figure S6).

## 4. Discussion

Planarians are resilient to low-dose 5FU treatment, and *DjsoxP*-*1^high^* sigma-class neoblasts [5,20] reappear after initial sterilization and repopulate the planarian body. In contrast, *DjpiwiA*- and *DjCIP29*-positive cells reduce in number slower than *DjsoxP-1^high^* cells, never completely disappear, and finally repopulate the planarian body. What happens to *DjsoxP-1^high^* cells early after 5FU treatment? According to our results, we hypothesize that SDT-600 treatment immediately blocks entrance in the S phase of the cell cycle, probably thanks to 5FU primary activity in inhibiting thymidylate synthase enzyme and thus producing dTTP shortage, which, in turns, block DNA synthesis [31]. In the following days, probably as a consequence of 5FU depletion (5FU is washed out 24 h after treatment) and increased levels of thymidylate synthase, a common cellular resistance mechanism described following short-term 5FU treatment [32], cells massively enter the S phase and BrdU incorporation exponentially increases in a sort of synchronized process. At this point, the still high dUTP/dTTP ratio most probably leads to frequent miss-incorporation of dUTP into the de-novo-synthesized DNA strand [33]. According to the literature, nucleotide unbalance and miss-incorporation produce a condition in which the replication fork progression slows down, producing fork pausing and instability, which, in turn, trigger an S-phase arrest [34] and unfinished DNA replication, thus activating a DNA damage response to avoid entry into mitosis with under-replicated or damaged chromosomes [35]. Indeed, following a massive S-phase entrance, the number of planarian mitotic cells remains low, leading us to hypothesize that the majority of stem cells remain blocked in the S phase of the cell cycle, following SDT-600 treatment. The few mitotic cells always detectable in the animal body of treated organisms might represent slow-cycling/G2-arrested cells, such as the TRSC [7] that asynchronously enter mitosis to produce post-mitotic progenies. Planarian stem cells arrested in the S phase have enough time to deal with DNA damage and do not die. On the contrary, a second 5FU treatment at the time of massive DNA synthesis causes unsustainable conditions, which prevents animal rescue. Thus, according to this scenario, the only possibility to explain, in the absence of cell death, the disappearance of *DjsoxP-1* expression and the permanence of *DjpiwiA* expression is to postulate that the S-phase slowdown triggers a change in the transcriptional profile in which the highly proliferating sigma-class neoblasts downregulate *DjsoxP-1*, so becoming *DjsoxP-1^low^*-committed cells. Later, most of the *DjsoxP-1^low^* /*DjPiwiA*-positive cells can be enrolled to undertake a physiological differentiation fate, losing *DjPiwiA* (and *DjP53*) expression, with the exception of the previously described TRSC [7]. This hypothesis satisfactorily correlates with the demonstration that neoblast specialization toward different fates occurs during the S phase of the cell cycle [5,36] and is confirmed by the permanence of an unmodified signal for *DjEGR-1*, *Djgata4/5/6*, and *DjNB.21.11.e*, which suggests that at least during the first 10 days after treatment, physiological cell turnover proceeds regularly.

According to this hypothesis, we can conclude that *DjsoxP-1^high^* cells are all proliferating; indeed, a 5FU treatment of 24 h is long enough to trigger S-phase slowdown and possibly S-phase arrest for all of them. On the contrary, the existence of a quiescent population of *DjsoxP-1^low^* stem cells that reactivate later after treatment is not consistent with our results, as these cells should, indeed, resist both the first and the second SDT-600 treatment (and eventually also higher doses), but this is not the case. However, in light of the recent discovery of a slow-cycling population of neoblasts, defined by low transcriptional activity (RNA^low^ neoblasts) [37], this possibility deserves further investigation.

Some of the *DjsoxP-1^low^*/S-phase-arrested cells produced by SDT-600 treatment probably succeed in repairing DNA damage, thus being again ready to divide and reach the G1 phase, in which *DjsoxP-1* expression is elicited again, transforming them into *DjsoxP-1^high^* cells. This possibility is consistent with the documented transcriptional changes during the neoblast cell cycle [36]. However, their reactivation is not an automatism and is probably subordinated to strong local signals to neutralize all the checkpoints imposed by the DNA damage response. In intact animals, according to the prominent reactivation of *DjsoxP-1* expression and proliferative activity at the ventral side of the body, we can hypothesize that local signals are released by ventral tissues that without a proper cell turnover start to age. This possibility will explain the about 20-day delay between treatment and brand-new *DjsoxP-1* expression. For example, we can postulate that the ventral epidermis without proper turnover might be one of the first tissues that loses its integrity, giving rise to a kind of extended-healing wound (H-wound), a wound in a no-missing tissue context. On the contrary, in regenerating animals, following amputation, a canonical regenerative wound (R-wound), a wound in a missing-tissue context, is produced. As a consequence, *DjsoxP-1* reactivation and proliferative activity are observed close to the wound site in both dorsal and ventral sides of regenerating animals. Several literature data demonstrate that in planarians, a common transcriptional response is initially activated after R- and H-wounds [38,39] and the potent waves of molecular signaling might be responsible for *DjsoxP-1* reactivation. In the case of an R-wound, novel *DjsoxP-1^high^* cells that appear at the wound surface might be, in part, involved in proliferative activity, their expression pattern being correlated with rare sparse mitoses detectable close to the wound site. However, most of the few mitoses detectable following cutting are located in the dorsal midline cord that in the special SDT-600 condition promptly respond to wounding stimuli and evidently activate cell division. However, both novel *DjsoxP-1^high^* cells as well as the proliferation of dorsal midline cord cells are not able to rescue the regenerative process, which completely fails. This is probably because *DjsoxP-1^high^* cells, which are theoretically able to sustain regeneration onset [5], are still limited in number, while TRSC are slow-cycling progeny unable to perform multiple rounds of cell division and self-renewal [7]. Failure in regeneration persists for a long period, also after *DjsoxP-1* and *DjpiwiA* expression is restored in both ventral and dorsal sides of the animal. The reason for such delay in restoring the regenerative potential is that at the time when the number of stem cells consistently increases (from 22 to 36 days after treatment), body tissues are highly compromised, as documented by the analysis of tissue sections and differentiated tissue markers. For example, dorsal clusters of *Djnos*-positive testis progenitors strongly reduce from 22 to 29 days after treatment and reappear well organized, as for controls, around day 32 after treatment. Under these generally compromised conditions, the cross talk between differentiated tissue and the stem cell compartment is probably impaired and not efficacious in eliciting regeneration onset. Indeed, in this context, extensive R-wounds worsen animal survival, leading to the death of most of them. *Djnos*-positive cells disappear after high-dose X-ray treatment [21,40] and are reduced later than *DjMCM2*-, *Djpum*-, and *DjCIP29*-positive cells after low-dose X-ray treatment [15]. These features led us to think that *Djnos* might be expressed in a subset of proliferating cells or that neoblasts may give rise to short-lived germ cells in asexual planarians [40]. Our data demonstrate the resistance of *Djnos*-positive cells to 5FU treatment, confirming that these cells, despite being highly positive for the planarian PCNA protein, are arrested in the cell cycle and unable to incorporate BrdU [21], thus not passing through the S phase, and are not damaged by dUTP miss-incorporation following 5FU treatment. Moreover, their late disappearance after treatment also led us to exclude that *Djnos*-positive cells are short-lived germ cells. Intriguingly, during the repopulation process, novel cells at the ventral side of the animal express *Djpiwi1*, a gene that under physiological conditions is only detectable in the dorsal midline cord [13]. *Djpiwi1* expression at the ventral side is transient. *Djpiwi1* function is unknown, but its transient activation during the repopulation process has been already demonstrated during neoblast repopulation that occurs in low-dose irradiated animals [15], thus again demonstrating that deep transcriptional modification occurs along with the rescue process.

Stem cell repopulation processes observed after low-dose X-ray exposure and SDT-600 5FU treatment share several common aspects, such as the reappearance of stem-cell-marker-positive cells and proliferative activity at the ventral side of the animal, in proximity to the nervous system [15]. However, the two treatments also show important distinctive features in terms of timing and resilient cells. This is probably the consequence of the different modalities of action of X-ray and 5FU. In the first case, wide DNA damage is produced in cells independently from the cell cycle phase. Thus, it is possible that all the undifferentiated cells respond to high DNA damage, inducing a massive cell death process, creating in a few days a shortage in the stem cell number in a healthy body with still intact tissues. Consequently, in accordance with the cross talk between differentiated and stem cell compartments, signals are released to push remnant stem cells to highly proliferate and repopulate the body. In the case of SDT-600 5FU treatment, only highly proliferating cells are damaged by the treatment and the DNA damage is enough to activate a DNA response, producing cell cycle arrest and not cell death. Therefore, stem cells change their transcriptional profile, but they are still there and in a first phase differentiate, accounting for tissue demand. Later, stem cell shortage progresses slowly and gradually parallel to tissue aging, up to a critical point, when some stem cells are pushed to reacquire proliferative self-renewing properties, engaging in a fight for animal survival. Our data reinforce the concept that it is wrong to think that neoblasts are categorizable into a strictly organized hierarchy of cells with specific transcriptional profiles and established differentiation potentiality and fate [36]. Although a progressive cell determination path of stem cells has been documented under physiological conditions [1,5,22], novel findings suggest that no known neoblast class is uniquely pluripotent and neoblasts from multiple classes can be clonogenic [36]. This might be further emphasized under challenging conditions, in which neoblasts in the earlier steps of commitment (or even early post-mitotic cells) can modulate their expression profile, reacquiring a wider differentiative potential. 

## Supplementary Materials

The following are available online at https://www.mdpi.com/article/10.3390/biom11070949/s1, Figure S1: *DjsoxP-1* expression visualized by whole-mount in situ hybridization in an intact untreated control and in an SDT-6000 animal sacrificed at different time points after treatment in dorsal (d) view. Figure S2: *DjsoxP-1* expression visualized by whole-mount in situ hybridization. (A) Representative image of *DjsoxP-1* expression in an intact SDT-600 animal sacrificed 22 days after treatment (ventral view). (B) Magnification of the region boxed in (A). Scale bar corresponds to 500 µm in (A) and 250 µm in (B). Figure S3: *DjPiwiA* expression visualized by whole-mount in situ hybridization. Representative images of *DjPiwiA* expression in intact SDT-600 animals sacrificed 25 days after treatment (ventral view). Scale bar corresponds to 500 µm in (A,B) and 380 µm in (C). Figure S4: Cell cycle analysis. (A) Representative plots of cytofluorimetric DNA content quantification. (B) Graph depicting the mean percentage ± SD of cellular distribution in G1-S-G2 cell cycle phases. Figure S5: Analysis of *DjsoxP-1* expression after single and double 5FU treatment. Representative images of *DjsoxP-1* in situ hybridization in 2-day-old regenerating control and treated animals sacrificed 15 days after the first 5FU treatment. Scale bar corresponds to 500 µm. Figure S6: Direct Red 80-/Fast Green-stained sections obtained from hybridized specimens. Images on the right are magnifications of the boxed region depicted in the corresponding images on the left. Scale bars correspond to 100 µm (for low magnification) and 25 µm (for high magnification). Table S1: (A) Distribution of 5FU specimens with respect to *DjsoxP-1* signal intensity vs control specimens 7 and 10 days after treatment. MGV=mean grey value; (B) Distribution of 5FU specimens with respect to *DjsoxP-1* expression pattern 22 days after treatment.
