# Supplementary material for: Sub-Lethal 5-Fluorouracil Dose Challenges Planarian Stem Cells Promoting Transcriptional Profile Changes in the Pluripotent Sigma-Class Neoblasts
Source: Biomolecules. 2021 Jun 26;11(7):949. doi: 10.3390/biom11070949 (PMC8301986; doi:10.3390/biom11070949)
Supplement: Supplementary file 1 [file biomolecules-11-00949-s001.zip › SUPPLEMENTARY FIGURES/Table S1.pdf]

Table S1

A) Distribution of 5FU specimens with respect to *DjsoxP-1* signal intensity *vs* control specimens 7 and 10 days after treatment. MGV=mean grey value

	**Number of analyzed specimens**	**% of 5FU animals with a MGV lower than the mean of control MGV**	**% of 5FU animals with a MGV lower than the half of the mean of control MGV**
***DjsoxP-1* expression level at day 7 after treatment**	18 specimens from 3 independent experiments	83%	55%
***DjsoxP-1* expression level at 10 seven after treatment**	18 specimens from 3 independent experiments	94%	72%

B) Distribution of 5FU specimens with respect to *DjsoxP-1* expression pattern 22 days after treatment

	**Number of analyzed specimens**	**Absent expression**	**Restricted pattern (similar to figure 1B)**	**Wider pattern (similar to figure S2)**
***DjsoxP-1* expression pattern at day 22 after treatment**	50 specimens from 5 independent experiments	17%	32%	51%

C) Distribution of 5FU specimens with respect to *DjsoxP-1* expression pattern 29 days after treatment

	**Number of analyzed specimens**	**Ventral signal restricted pattern (similar to figure 1B)**	**Ventral signal wider pattern (similar to figure S2)**	**Ventral signal widely extended pattern**	**Ventral and dorsal signal**
***DjsoxP-1* expression pattern at day 29 after treatment**	50 specimens from 3 independent experiments	4%	4%	50%	42%
