# Supplementary figures and images for: Sub-Lethal 5-Fluorouracil Dose Challenges Planarian Stem Cells Promoting Transcriptional Profile Changes in the Pluripotent Sigma-Class Neoblasts
Source: Biomolecules. 2021 Jun 26;11(7):949. doi: 10.3390/biom11070949 (PMC8301986; doi:10.3390/biom11070949)

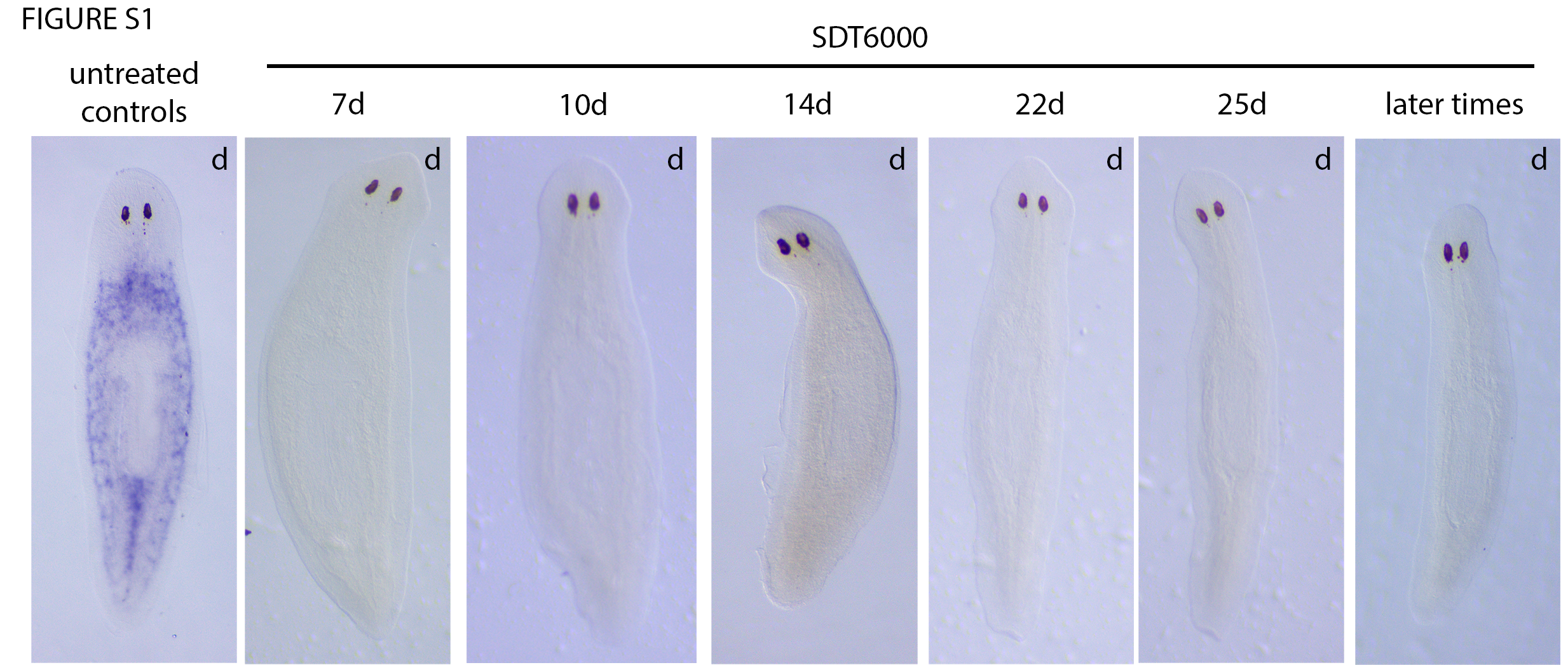

Supplement: Supplementary file 1 [file biomolecules-11-00949-s001.zip › SUPPLEMENTARY FIGURES/FIGURE S1.tif]

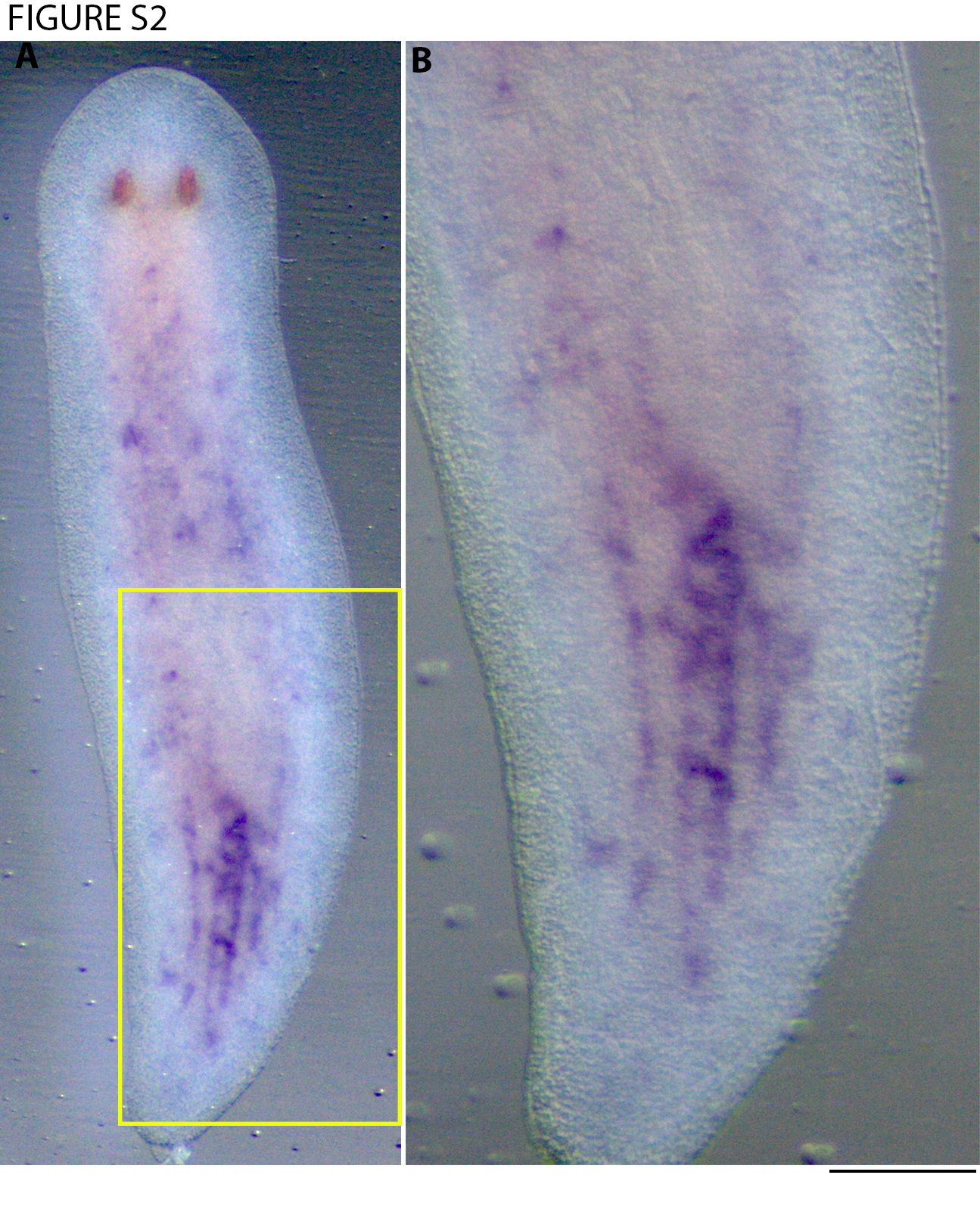

Supplement: Supplementary file 1 [file biomolecules-11-00949-s001.zip › SUPPLEMENTARY FIGURES/FIGURE S2.tif]

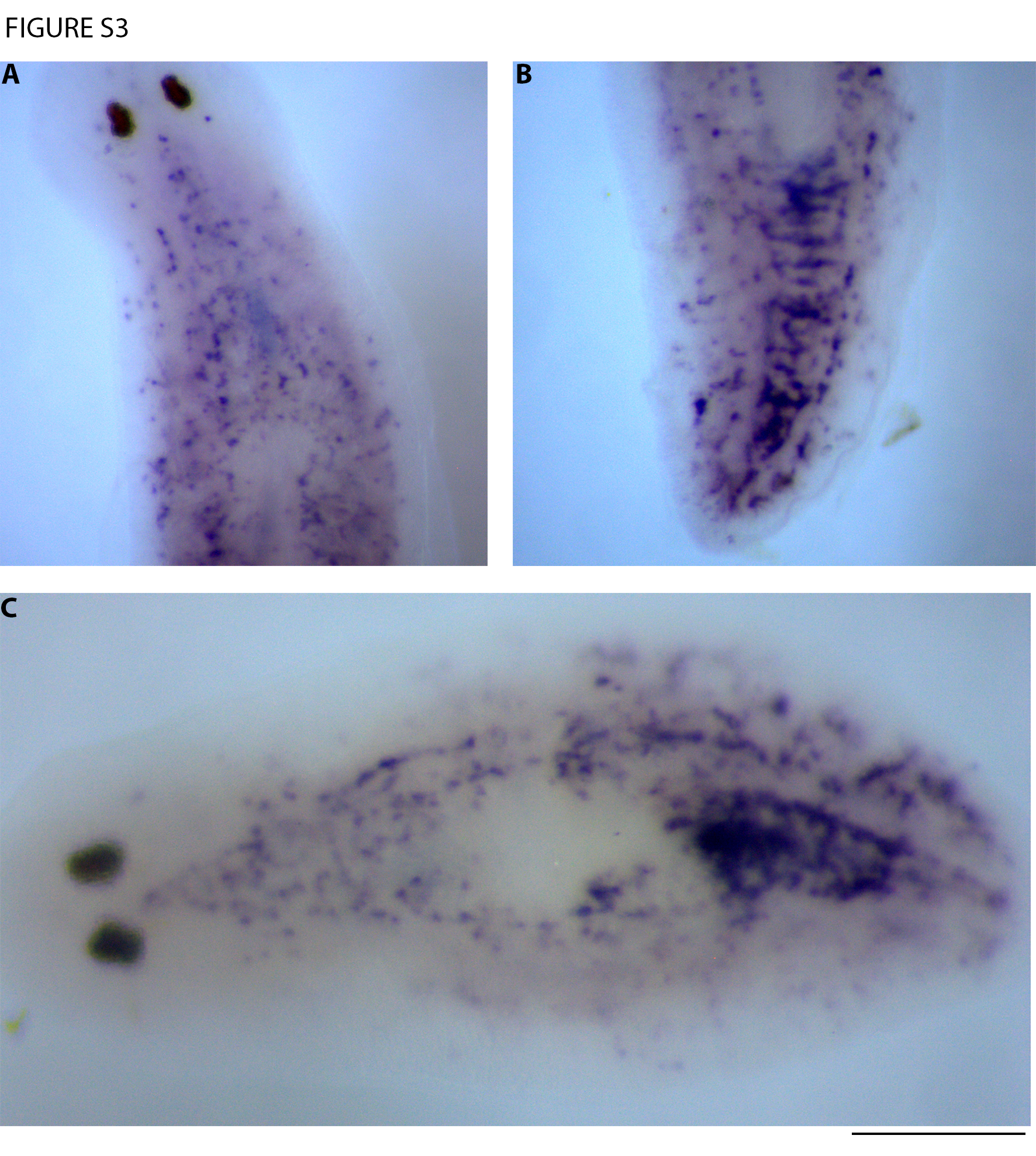

Supplement: Supplementary file 1 [file biomolecules-11-00949-s001.zip › SUPPLEMENTARY FIGURES/FIGURE S3.tif]

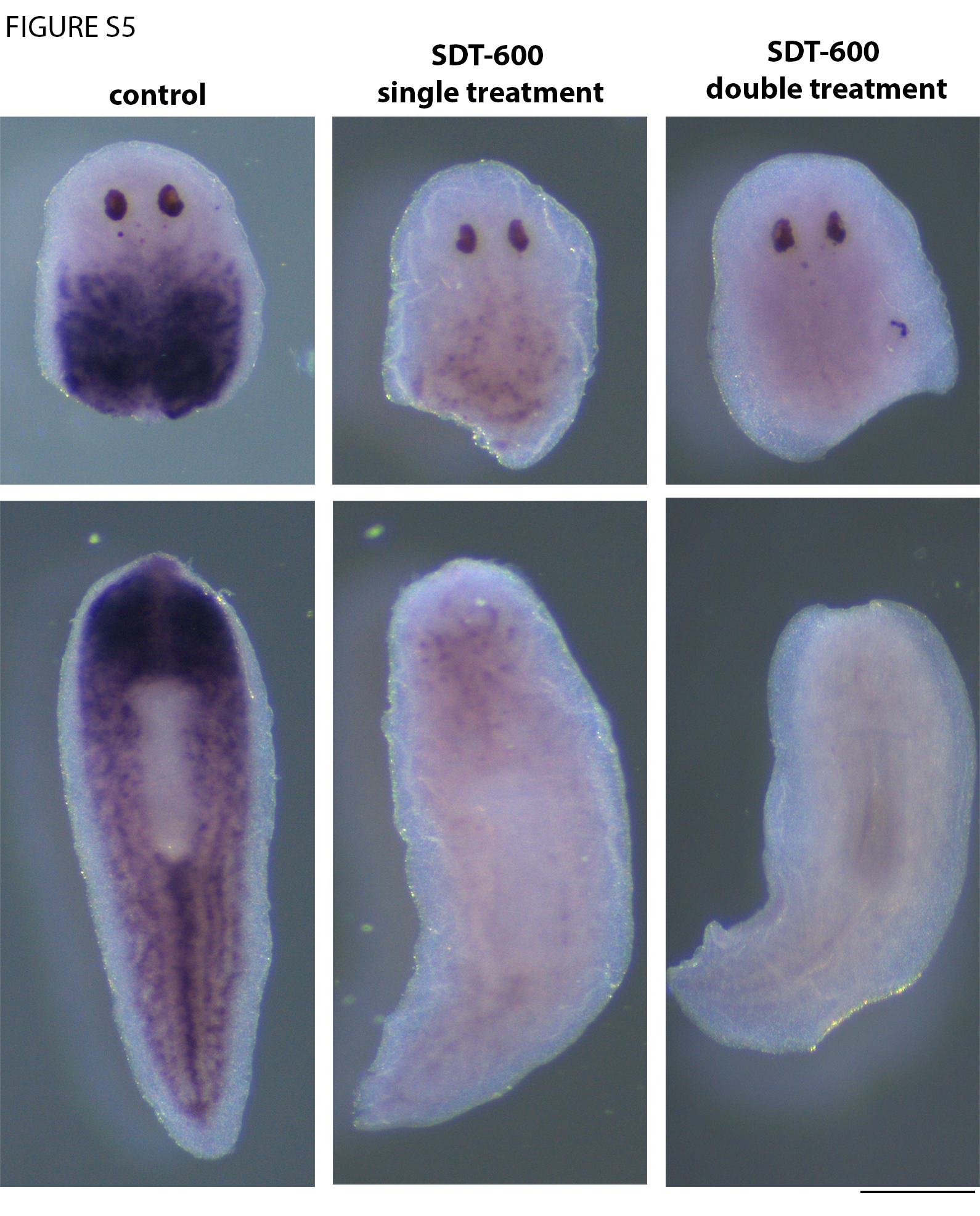

Supplement: Supplementary file 1 [file biomolecules-11-00949-s001.zip › SUPPLEMENTARY FIGURES/FIGURE S5.tif]

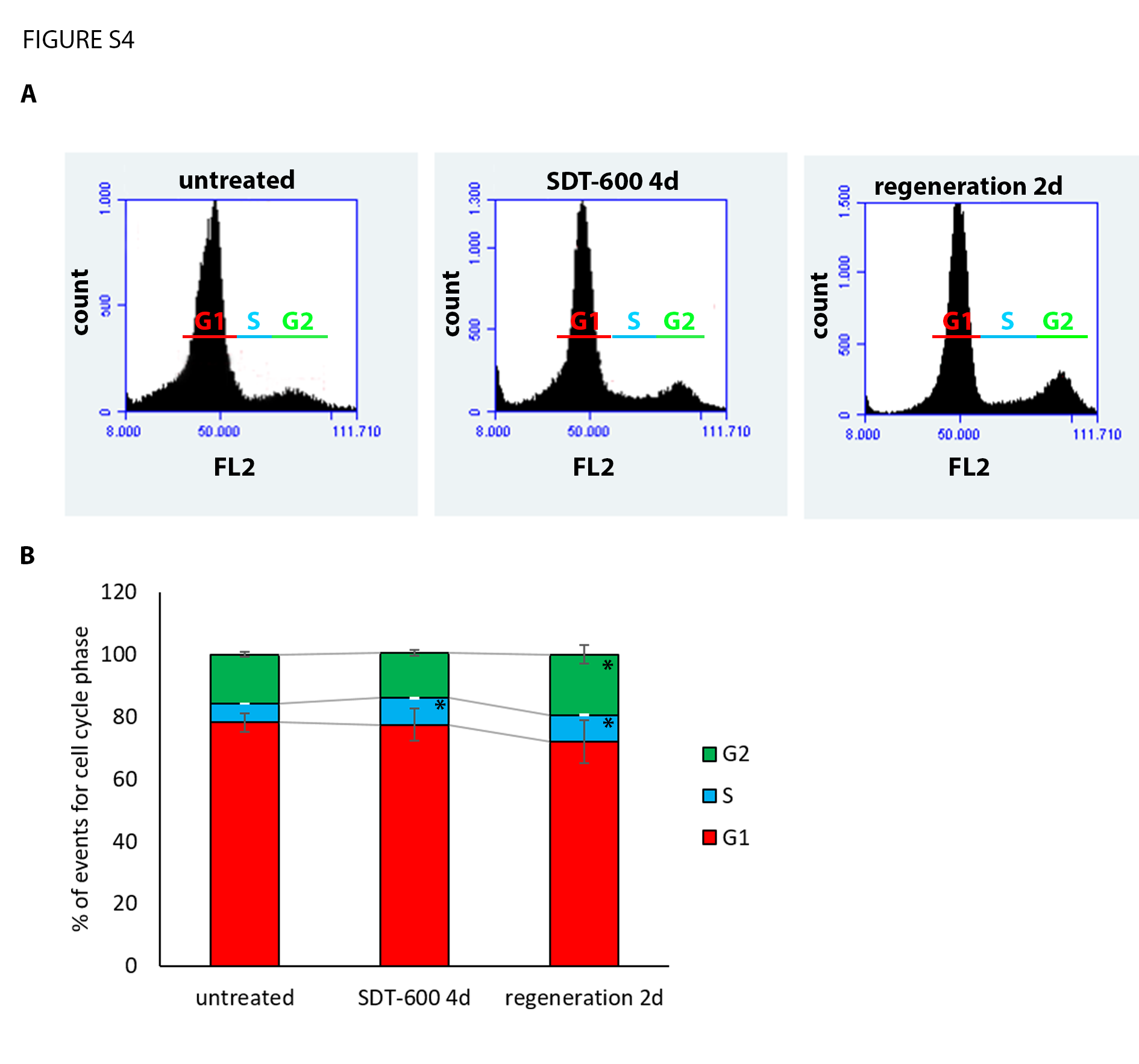

Supplement: Supplementary file 1 [file biomolecules-11-00949-s001.zip › SUPPLEMENTARY FIGURES/Figure S4.tif]

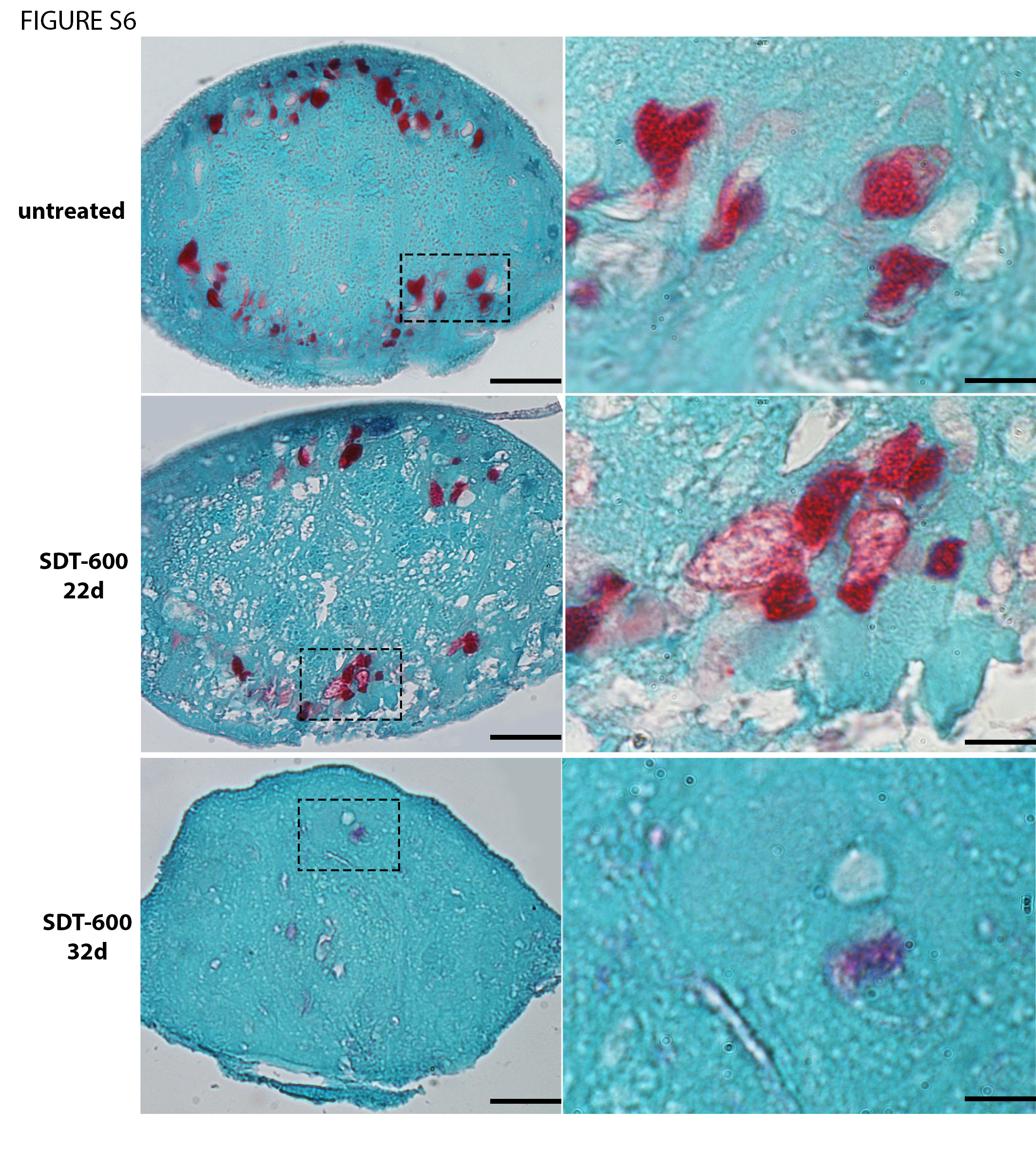

Supplement: Supplementary file 1 [file biomolecules-11-00949-s001.zip › SUPPLEMENTARY FIGURES/Figure S6.tif]
